# Should highly active antiretroviral therapy be prescribed in critically ill HIV-infected patients during the ICU stay? A retrospective cohort study

**DOI:** 10.1186/1742-6405-9-27

**Published:** 2012-09-28

**Authors:** Agnes Meybeck, Lydie Lecomte, Michel Valette, Nicolas Van Grunderbeeck, Nicolas Boussekey, Arnaud Chiche, Hugues Georges, Yazdan Yazdanpanah, Olivier Leroy

**Affiliations:** 1 Service de Réanimation et Maladies Infectieuses, Hôpital de Tourcoing, 135 rue du Président Coty, Tourcoing BP 619, France; 2 Service des Maladies Infectieuses et du Voyageur, Hôpital de Tourcoing, 135 rue du Président Coty, Tourcoing BP 619, France; 3 Service de Réanimation et Maladies Infectieuses, Centre Hospitalier Dron, 128 rue du Président Coty, Tourcoing 59200, France

**Keywords:** HIV, Intensive care, HAART

## Abstract

**Background:**

The impact of highly active antiretroviral therapy (HAART) in HIV-infected patients admitted to the intensive care unit (ICU) remains controversial. We evaluate impact of HAART prescription in HIV-infected patients admitted to the ICU of Tourcoing Hospital from January 2000 to December 2009.

**Results:**

There were 91 admissions concerning 85 HIV-infected patients. Reasons for ICU admission were an AIDS-related diagnosis in 46 cases (51%). Fifty two patients (57%) were on HAART at the time of ICU admission, leading to 21 immunovirologic successes (23%). During the ICU stay, HAART was continued in 29 patients (32%), and started in 3 patients (3%). Only one patient experienced an adverse event related to HAART. Mortality rate in ICU and 6 months after ICU admission were respectively 19% and 27%. Kaplan-Meier estimates of the cumulative unajusted survival probability over 6 months were higher in patients treated with HAART during the ICU stay (Log rank: p = 0.04). No benefit of HAART in ICU was seen in the adjusted survival proportion at 6 months or during ICU stay. Prescription of HAART during ICU was associated with a trend to lower incidence of new AIDS-related events at 6 months (respectively 17% and 34% with and without HAART, p = 0.07), and with higher incidence of antiretroviral resistance after ICU stay (respectively 25% and 7% with and without HAART, p = 0.02).

**Conclusions:**

Our results suggest a lower death rate over 6 months in critically ill HIV-infected patients taking HAART during ICU stay. The optimal time to prescribe HAART in critically ill patients needs to be better defined.

## Background

Highly active antiretroviral therapy (HAART) has increased the life expectancy of patients who are infected with the human immunodeficiency virus (HIV) and has reduced the incidence of illnesses associated with the acquired immunodeficiency syndrome (AIDS). HIV-infected adults with CD4 cell count greater than 500 cells/mm3 on long-term combination antiretroviral therapy reach same mortality rates as the general population [1]. Survival of HIV-infected patients admitted to the intensive care unit (ICU) has been evaluated in a number of studies [2-6]. Most of these studies have shown improved survival since the late 1990s for HIV infected patients, associated with the advent of HAART. But whether or not the improved survival is dependent of the use of HAART remains controversial [5-7]. Changing practices of ICU care and decrease of AIDS-related admissions may explain survival improvement [2,8].

Very few previously published studies have reported data on the use of HAART in the ICU [9-11]. There are no randomized clinical trials and no consensus guidelines to assist in decisions regarding the use of HAART in the ICU [11]. Early prescription of HAART in ICU might decrease patient morbidity and mortality by restoring pathogen specific immune responses and speeding immune reconstitution. On the other hand, prescription of HAART in critically ill patients might result in higher morbidity and mortality by increasing toxicity of treatment, increasing drug-drug interactions, increasing the frequency of immune reconstitution and inflammatory syndrome (IRIS).

We conducted a retrospective cohort study of all consecutive cases of HIV-infected patients admitted to the ICU from January 2000 to December 2009 at Tourcoing Hospital. The aim of this study was to evaluate the impact of HAART prescription prior to ICU and during ICU stay.

## Methods

### Patients and hospital setting

We conducted a retrospective cohort study of all consecutive cases of HIV-infected patients admitted to the 16-bed ICU of Tourcoing Hospital from January 2000 to December 2009. As it was an observational retrospective study, in accordance with French law, neither approval of the ethics committee nor informed consent was required. If patients were admitted to the ICU more than one time during any hospitalisation, only data from admissions separated by > 90 days were considered independent events and were included in the analysis.

### Data collection and baseline data

Data on HIV infection and on ICU stay were obtained retrospectively, using case-notes and computer database. Data obtained included age, sex, race, the indication(s) for ICU admission, underlying clinical conditions, presence of comorbidities, severity of illness at ICU admission. Admission was considered AIDS-related if the main admission diagnosis was an AIDS-defining condition (1993 Centers for Disease Control Classification System for HIV-Infected Adults) [12]. The underlying clinical conditions were classified according to the criteria proposed by McCabe and Jackson in 3 categories: non fatal (5 year survival not affect by underlying disease), ultimately fatal (not expected to survive more than 5 years), and rapidly fatal (not expected to survive more than 1 year) [13]. The severity of illness at the time of ICU admission was assessed by SAPS II and Glasgow Coma Score [14,15]. The SAPS II includes 12 physiology variables including Glasgow Coma Score, and age, type of admission (scheduled surgical, unscheduled surgical, or medical), and three underlying disease variable (acquired immunodeficiency syndrome, metastatic cancer, and hematologic malignancy). Neurological and mental status was stratified according to Glasgow Coma Score. The following therapeutic modalities in ICU were recorded: invasive or noninvasive mechanical ventilation, hemodialysis or hemofiltration, and use of vasoactive drugs.

The following data on HIV-infection were recorded for each case at ICU admission: risk factor for HIV, HIV disease staging according to CDC Classification System for HIV-Infected Adults (Category A: asymptomatic, acute HIV, or persistent generalized lymphadenopathy; Category B: symptomatic conditions not A or C; Category C: AIDS-indicator conditions), CD4 cell count and plasma HIV viral load if available within 6 months of admission. The mean time between determination of CD4 cell count and plasma HIV viral load and admission in ICU was 32 days. During ICU stay, we recorded new AIDS-defining event and IRIS occurence. IRIS was defined as a paradoxical deterioration in clinical status or laboratory findings after the initiation of antiretroviral therapy without another attribuable cause.

The following data on antiretroviral therapy were recorded: HAART prior and during ICU stay. We collected regimen, duration and adverse effects. HAART was defined as a combination of 3 or more antiretroviral drugs belonging to at least 2 classes among the following: protease inhibitors (PIs), nucleoside reverse transcriptase inhibitors (NRTIs), and nonnucleoside reverse transcriptase inhibitors (NNRTIs). No other antiretroviral combination than HAART was prescribed in our cohort.

After the ICU stay, the following data were recorded: CD4 cell count and viral load at 3, 6, and 12 months after ICU admission, occurence of a new AIDS defining event, occurence of IRIS, change in HAART prescription, emergence of antiretroviral-resistance at any time during a 12 months period following ICU admission.

### Outcome measures

Our primary outcomes of interest were in ICU and 6-month mortality. Secondary outcomes of interest were the occurrence of a new AIDS related event, immunovirological response, adverse event of antiretroviral therapy, emergence of resistance. Immunovirological success was defined as viral load < 200 copies/ml and CD4 > 200 cells/mm^3^.

### Statistical analyses

Analyses were performed to identify predictive factors of ICU and 6-month mortality. All collected variables concerning underlying disease, severity of illness, care in ICU, and HIV-infection were analysed. Categorical variables were compared using Chi-square test or Fisher’s exact test when Chi-square was not appropriate. Continuous variables were compared using Student’s *t* test or Mann–Whitney test when appropriate. All variables attaining an α value of 0.05 were included in a multiple logistic regression analysis model with a stepwise selection of variables to identify predictive factors of ICU and 6-month mortality. Variables that originate a multicolinearity were eliminated. The quality of the model was evaluated through its measures of sensitivity and specificity with establishment of a ROC curve. Kaplan Meier survival analysis was used to estimate cumulative mortality over 6 months. Log rank test was used to compare differences in survival between two groups (no HAART use in ICU vs HAART use in ICU). Differences between groups were considered to be significant for variables yielding a p value ≤ 0.05.

## Results

### Demographic and clinical characteristics

There were 91 ICU admissions concerning 85 HIV-infected patients. Demographic and clinical characteristics of our patients are shown Table 1. The majority of the ICU cohort consisted of men (n = 61, 67%), and was ever diagnosed with AIDS (n = 69, 76%). Patients were equally likely to be heterosexual (n = 31, 34%), or homosexual (n = 31, 34%). Median CD4 cell count at ICU admission was 112/mm^3^ [1–935]. The ICU admission represented the initial HIV diagnosis for 19% of the cases. Respiratory failure and neurologic disorder were the most frequent reasons for ICU admission (51% and 27% respectively). The ICU admissions were motivated by AIDS-associated diagnosis in 46 patients (51%). HAART adverse events led to ICU admission in 4 patients. Mechanical ventilation was needed in 45 patients (49%) for a mean duration of 5 ± 10 days. The mean length of ICU stay was 8 ± 10 days.

**Table 1:** Characteristics of the patients and clinical data depending on HAART use during ICU stay*

**Characteristics and clinical data**	**Total (n = 91)**	**without HAART (n = 59)**	**with HAART (n = 32)**	**p**
*Characteristics*				
Age (in years)	42 ± 10	42 ± 9	43 ± 11	0.98
Sex (M/F)	61/30	40/19	21/11	0.99
SAPS II on admission	47 ± 20	48 ± 21	46 ± 17	0.83
Glasgow score	12 ± 4	12 ± 5	13 ± 4	0.24
Main reasons for ICU admission				
Respiratory	46 (51)	28 (47)	18 (56)	0.51
Neurologic	25 (27)	16 (27)	9 (28)	0.99
Shock	10 (11)	6 (10)	4 (13)	0.99
Others	10 (11)	9 (15)	1 (3)	0.09
*HIV infection*				
Initial HIV diagnosis	17 (19)	16 (27)	1 (3)	**0.004**
Stage C	69 (76)	43 (73)	26 (81)	0.85
CD4 count at admission (cells/mm^3^)	112 [1–935]	100 [2–935]	161 [1–822]	0.68
Viral load at admission (log10)	3.38 ± 1.60	3.86 ± 1.51	2.63 ± 1.45	**0.0005**
AIDS related diagnosis at ICU admission	46 (51)	33 (56)	13 (41)	0.25
Opportunistic infections				
Pneumocystis jirovecii	12	10	2	0.2
Toxoplasmosis	7	5	2	0.99
Mycobacterial infections	7	4	3	0.69
Pneumonia	3	2	1	0.99
Others	5	3	2	0.99
*Care in ICU*				
Mechanical ventilation (MV)	45 (49)	29 (49)	16 (32)	0.87
Duration of ventilation (days)	5 ± 10	5 ± 11	5 ± 9	0.81
Renal replacement	7 (8)	3 (5)	4 (13)	0.33
Vasoactive drugs	22 (24)	15 (25)	7 (22)	0.99
Duration of vasoactive drugs (days)	1 ± 2	1 ± 2	1 ± 2	0.99
ICU length of stay (days)	8 ± 10	8 ± 11	8 ± 9	0.43
Death in ICU	17 (19)	12 (20)	5 (16)	0.78

*Data are presented as No. (%) or mean ± SD, except for CD4 cell count which are expressed as median [range].

Patients who received HAART in ICU were similar to those who did not with respect to age, gender, mean SAPS II, and Glasgow score. Patients who received HAART in ICU were less likely to be ignorant of their HIV infection (3% vs 27%, p = 0.004) and had significantly lower mean log viral load (2.63 ± 1.45 log copies/ml vs 3.86 ± 1.51 log copies/ml, p = 0.0005). Median CD4 cell count at admission was similar in both groups.

### Risk factors for mortality

Mortality rate in ICU, and 6 months after ICU admission were respectively 19% and 27%. Results for univariate analysis for ICU and 6-month outcomes are presented respectively in Table 2 and Table 3.

**Table 2:** Univariate predictors of ICU survival*

**Characteristics**	**Non survivors n = 17 (19)**	**Survivors n = 74 (81)**	**p**
*Characteristics at ICU admission*			
Age (in years)	40 ± 9	43 ± 10	0.13
Sex (M/F)	7/10	23/51	0.57
Glasgow score	8 ± 5	13 ± 4	**0.0003**
*HIV infection*			
Initial HIV diagnosis	7 (37)	10 (14)	**0.015**
Stage C	13 (76)	56 (76)	0.99
CD4 count at admission (cells/mm3)	120 ± 98	206 ± 209	0.38
AIDS related diagnosis at ICU admission	11 (65)	35 (47)	0.28
*Care in ICU*			
Mechanical ventilation	17 (100)	28 (38)	**<0.0001**
Duration of ventilation (days)	12 ± 13	4 ± 9	**<0.0001**
Vasoactive drugs	5 (29)	8 (11)	0.06
Duration of vasoactive drugs use (days)	2.5 ± 2.7	0.7 ± 1.7	**<0.0001**
*Use of HAART*			
HAART at ICU admission	6 (38)	46 (62)	0.09
^**§**^ immunovirological success at ICU admission	2 (12)	19 (26)	0.34
HAART during ICU stay	5 (29)	27 (36)	0.78

*Data are presented as No. (%) or mean ± SD. ^§^Immunovirological success was defined as viral load < 200 copies/ml and CD4 > 200 cells/mm^3^.

**Table 3:** Univariate predictors of 6-month survival*

**Characteristics**	**Non survivors n = 25**	**Survivors n = 55**	**p**
*Characteristics at ICU admission*			
Age	40 ± 9	42 ± 9	0.19
Sex (M/F)	8/17	19/36	0.99
SAPSII on admission	55 ± 25	42 ± 16	**0.02**
Glasgow on admission	10 ± 5	13 ± 4	**0.05**
*HIV infection*			
Initial HIV diagnosis	8 (32)	6 (11)	**0.03**
Stage C	20 (80)	43 (78)	0.99
CD4 count at admission (/mm3)	154 ± 201	215 ± 198	0.21
AIDS related diagnosis at ICU admission	16 (64)	24 (44)	0.15
*Care in ICU*			
Mechanical ventilation	19 (76)	20 (36)	**0.0015**
Duration of ventilation (days)	10 ± 13	3 ± 8	**0.0001**
Vasoactive drugs	6 (24)	5 (9)	0.09
Duration of vasoactive drugs use (days)	1.7 ± 2.5	0.6 ± 1.3	**0.006**
*Use of HAART*			
HAART at ICU admission	11 (44)	35 (64)	0.14
immunovirological success at ICU admission	3 (12)	17 (31)	0.096
HAART during ICU stay	9 (36)	21 (38)	0.99

*Data are presented as No. (%) or mean ± SD.

By univariate analysis, the factors associated with ICU mortality were high SAPS II score, low Glasgow score, use of mechanical ventilation, prolonged duration of mechanical ventilation, and of catecholamine prescription. Patient with undiagnosed HIV were more likely to die in ICU. By univariate analysis, the factors associated with 6-month mortality were similar to those associated with ICU mortality. Neither history of HAART use prior to ICU admission, nor use of HAART during ICU stay was associated with ICU and 6-month survival.

**Figure 1:**
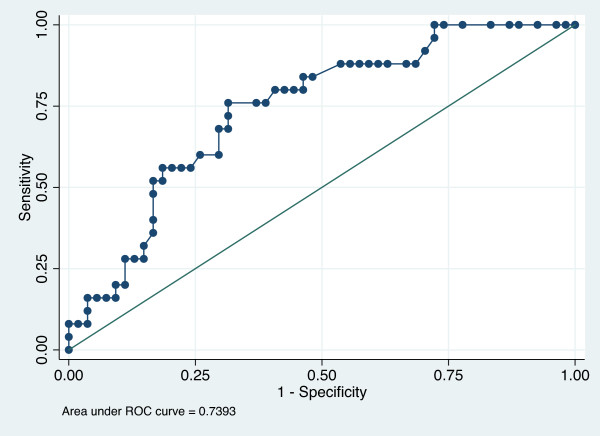
**ROC curve evaluating quality of the model including SAPS II and AIDS-related diagnosis to determine 6-month mortality risk.** The quality of the multiple logistic regression analysis model was evaluated through its measures of sensitivity and specificity with establishment of a ROC curve. Aera under the ROC curve was 0.7393.

Results for multivariate analysis for ICU and 6-month mortality are presented respectively in Table 4 and Table 5. By multivariate analysis, the only factor independently associated with ICU mortality was SAPS II score (OR = 1.05 per point; 95%CI: 1.02-1.08; p = 0.001). SAPS II score and AIDS-associated diagnosis were independently associated with 6-month mortality (respectively OR = 1.05 per point; 95%CI: 1.01-1.06; p = 0.001 and OR = 2.9, 95%CI: 1.01-8.31; p = 0.04). The quality of the model has been evaluated through its measures of sensitivity and specificity with establishment of a ROC curve, the area under the curve being 0.739 (Figure 1).

**Table 4:** Multivariate predictors of ICU survival*

**Variables**	**Adjusted OR (95% CI)**	**p value**
SAPS II on admission (per 1 point of increment)	1.05 (1.02-1.08)	0.001

*The retained variables for the multivariate analysis were those the adjustment of which had a medical or biological meaning, eliminating that originate a multicolinearity. Variables were retained when their effect had a P value less than 0.05.

**Table 5:** Multivariate predictors of 6-month survival*

**Variables**	**Adjusted OR (95% CI)**	**p value**
SAPS II on admission (per 1 point of increment)	1.05 (1.01-1.06)	0.001
AIDS related diagnosis	2.9 (1.01-8.31)	0.04

*The retained variables for the multivariate analysis were those the adjustment of which had a medical or biological meaning, eliminating that originate a multicolinearity. Variables were retained when their effect had a P value less than 0.05.

### Use of HAART

At the time of ICU admission, 52/91 (57%) of patients were on HAART, leading to only 21 (23%) immunovirological successes. A combination of two nucleoside/nucleotide reverse transcriptase inhibitor (NRTI) and one protease inhibitor (PI) was the most common prescribed regimen (n = 31/52, 59%).

During ICU stay, antiretroviral therapy was continued in 32 patients (35%), and initiated in ICU in 3 patients (3%). In patients already receiving antiretroviral therapy at ICU admission, HAART was continued in 26 patients (50%), modified in 3 patients (6%), stopped in 23 patients (44%). HAART interruption was motivated by a severe side effect in 4 patients. In 19 patients, no justification for HAART discontinuation was found in the patient chart. HAART was stopped despite immunovirological success in 9 patients. The 3 patients who changed HAART, replaced another boosted protease inhibitor by the combination lopinavir-norvir, available in liquid form. The median delay between ICU admission and HAART prescription was 0 day [0–38]. In case of HAART initiation in ICU, this delay was prolonged: 19 [2–38] days. The duration of HAART in ICU was 5 ± 4 days. Patients treated with HAART in ICU spent 76% of their ICU stay on HAART. The most commonly prescribed regimen in ICU was the combination of two NRTI and one PI (n = 24/32, 75%).

All patients who received HAART during ICU stay continued HAART after ICU stay. After ICU discharge, antiretroviral combination was modified in 8 patients, because of drug interaction in 3 patients, adverse event occurence in 2 patients, virological failure in one patient, addition of enfuvirtide in one patient. No motivation was found in one patient. HAART was initiated (n = 14) or reintroduced (n = 15) in patients not treated in ICU after a mean delay of 19 ± 22 days after ICU release. The most common prescribed regimen after ICU stay was the combination of two NRTI and one PI (n = 38/61, 62%).

### Impact of HAART on survival

**Figure 2:**
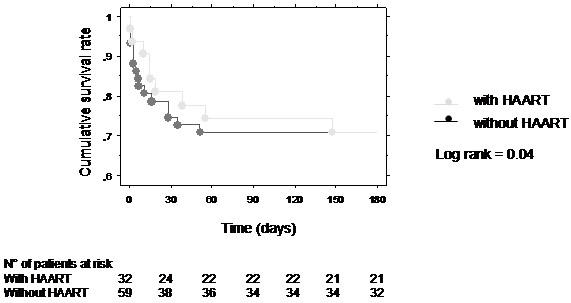
**The effect of the prescription of antiretroviral therapy in ICU on 6-month cumulative survival.** Kaplan-Meier estimates of the cumulative unajusted survival probability over 6 months were higher in patients with HAART (grey line) vs without HAART(black line) during the ICU stay (Log rank: p = 0.04).

The mortality rate was not significantly different at ICU discharge and at 6 months according to the use of HAART during the ICU stay: respectively 16% vs 20% with and without HAART at ICU discharge, and 28% vs 27% respectively with and without HAART at 6 months. Kaplan-Meier estimates of the cumulative unadjusted survival probability over 6 months were significantly higher in the group of patients treated with HAART during the ICU stay (Log rank test, p = 0.04) (Figure 2). But, no benefit of HAART was seen specifically in the adjusted survival proportion at 6 months or during ICU stay (Table 2 and Table 4).

### Impact of HAART on AIDS progression and immunovirologial response

During ICU stay, a new AIDS related event was diagnosed in 3 patients (9%) treated with HAART, and in 5 patients (8%) not treated with HAART. At 6 months, new AIDS related events tended to be less frequent in the group of patients treated with HAART in ICU, respectively 17% vs 34% with and without HAART (p = 0.07).

When comparing mean CD4 cell count, there was no significant difference between the two groups (with vs without HAART) at any time of the year following ICU admission. Median CD4 cell count at 12 months reached 1309 [365–2254] cells/mm^3^ and 385 [99–605] cells/mm^3^ respectively with and without HAART (p = 0.23). Mean log10 viral load was significantly lower at 3 and 6 months in patients treated with HAART while in ICU (2.06 ± 0.78 vs 3.16 ± 1.46 copies/ml with and without HAART at 3 months, p = 0.02, and 2.42 ± 0.68 vs 3.37 ± 1.36 copies/ml with and without HAART at 6 months, p = 0.03). One year after ICU admission, immunovirological success was achieved in 25% of patients treated with HAART and in 16% of patients not treated with HAART (p = 0.25).

A resistance to an antiretroviral molecule was diagnosed after ICU in 8 patients (25%) treated with HAART, and in 4 patients (7%) not treated with HAART (p = 0.02). A history of HAART prior to ICU and an immunovirologic failure at ICU admission were reported respectively in 12 and 5 patients diagnosed with antiretroviral resistance after ICU.

### Tolerance of HAART

In ICU, a side effect motivating interruption of HAART was reported in one patient, who suffered from severe anemia while treated with zidovudine. After ICU stay, 21 adverse events motivating interruption or change of HAART were reported after a mean duration of treatment of 107 ± 112 days. The incidence of adverse events after ICU was not different according to the timing of HAART prescription, during or after the ICU stay. An IRIS was diagnosed in 2 patients (2.2%). Both IRIS occured after ICU stay, respectively 25 days and 45 days after ICU admission. One patient received HAART in ICU. The other one began HAART the day he left the ICU, 7 days after his admission.

## Discussion

In our cohort of HIV-infected patients admitted to the ICU, the only independent predictor of ICU outcome was severity of acute illness assessed by SAPS II score at ICU admission. Two factors were independent predictors of decreased 6-month survival: SAPS II score and AIDS-associated diagnosis at ICU admission. Use of HAART during ICU stay improved unadjusted survival at 6 months. But no benefit was seen specifically in the adjusted survival proportion at 6 months or during ICU stay. Use of HAART during ICU stay tended to reduce AIDS progression. Tolerance of HAART in ICU was good. IRIS was rare. Prescription of HAART during ICU stay was associated with higher incidence of antiretroviral resistance after ICU stay.

In our cohort, risk factors for ICU mortality were consistent with factors previously identified in the literature; these relate to the extent of organ dysfunction rather than HIV-related characteristics [6,7,16]. Severity of acute illness assessed by various scores (APACHE-II score, SAPS-II), need for mechanical ventilation, or catecholamine predicted higher mortality rate in ICU [2,3,6,7,16,17]. Low serum albumin level has been associated with increased mortality rate in ICU in several studies [6,7,17,18].

In our series, AIDS-related disease at the time of ICU admission was associated with a higher 6-month mortality rate. Other previous reports demonstrated an impact on long term survival of AIDS-associated diagnosis in the ICU [2,6,17]. Morris A et al. reported that the patients with a non-AIDS-associated diagnosis had a dramatically improved median survival compared with those with an AIDS-associated diagnosis [6]. Khouli H et al. showed HIV-related illness to be a significant independent predictor of increased hospital mortality [17]. Among the HIV-related characteristics, the CD4 cell count may be a predictor of long-term outcome [19]. But, several authors, like us, did not report any significant relationship between the CD4 cell count and survival [4,6,20].

Concerning HAART prescription, almost two thirds of our patients were on HAART at the time of ICU admission. But only 23% of the patients have achieved immunovirological success. Prior HAART was continued in ICU in only 53% of the patients, and HAART was almost never initiated in ICU. By multivariate analysis, the use of HAART prior to ICU admission and during ICU stay did not independently affect ICU or 6-month survival. But the log rank test suggests a lower death rate over 6-months in patients treated with HAART in ICU. The discrepancy between log-rank curves and the multivariate analysis may be due to potential confounders. Patients without HAART in ICU were more likely to be ignorant of their HIV infection and tended to suffer more often from an opportunistic disease. This could influence the prognosis. Patients with HAART in ICU tended to experience fewer AIDS-associated diagnosis during the 6-month follow-up period. Improved immune function and increased CD4 cell count following HAART could explain the reduction of AIDS progression in case of HAART use in ICU. Published data that have assessed the impact of HAART in ICU are few. Several retrospective studies have reported an improvement of survival of HIV-infected patients admitted in ICU in the last decade, since the advent of HAART [2,4,6]. But the impact of HAART on survival is intringuing. The decrease in mortality could be a result of a change in the spectrum of admission diagnosis as non AIDS-associated diagnosis became more common and were associated with a better survival than AIDS-associated diagnosis. Powell K. et al. examined survival to hospital discharge of HIV-infected patients admitted to the ICU since the year 2000 [7]. They reported that survival for critically ill HIV-infected patients continues to improve in the current era of HAART. This improvement appeared to be independent of the use of HAART. Many advances in critical care practice (e.g., low tidal volumes for patients with respiratory failure, tight glycemic control, adjuvant corticosteroïds for certain conditions, early goal-directed therapy for sepsis) have been made across this last decade [21-23]. They could contribute to outcome improvement of HIV infected patients admitted in ICU.

None of these observational studies reported data on the prescription of HAART during the ICU stay. Only three retrospective studies have assessed the potential benefit associated with administrating HAART in the ICU. Morris et al. performed a retrospective study of 58 patients admitted to the ICU with *Pneumocystis jirovecii* between 1996 and 2001 [24]. They found that patients who were receiving HAART at the time of ICU admission or had HAART started during the ICU stay had a reduced mortality rate compared to those who did not receive HAART. Another retrospective study conducted in a Mexican ICU found HAART prior to admission or starting within one month after ICU discharge to be the major protective factor for death [9]. In their cohort, Croda et al. reported a significantly better survival of patients who began HAART during ICU stay compared to patients without HAART during ICU stay [10]. The potential benefit of HAART found in these three studies could reflect the impact of early prescription of HAART in the context of AIDS-defining diagnosis. An AIDS-defining diagnosis motivated ICU admission in 51% of patients in our cohort and in up to 80% of patients in the last study mentioned. But, in our population, only 19% of patients had not been diagnosed with HIV infection prior to their admission in ICU. A majority of our patients had history of HAART. The very small proportion of patients initiating HAART in our cohort could explain the lack of significant impact of HAART prescription on prognosis. Recently, Zolopa et al. conducted a prospective randomized study to compared immediate HAART (within two days of study enrollment) to deferred HAART (between week 6 and week 12) in HIV-patients diagnosed with acute AIDS-related opportunistic infection [25]. Early HAART resulted in less AIDS progression and death compared to deferred HAART. Concurrent treatment of the opportunistic infection and HIV might increase the frequency of IRIS and have a deleterious impact on outcomes. Incidence and consequences of IRIS vary by opportunistic infections and corticoid use [25-27]. In case of cryptococcal meningitis, early initiation of HAART resulted in increased rate of IRIS, and increased mortality [26]. In case of tuberculosis, HAART started during tuberculosis therapy resulted in lower mortality despite higher incidence of IRIS [27]. In the setting of the opportunistic infections observed in our cohort, most particularly *Pneumocystis* pneumonia, IRIS occurence was rare. Only one previous study conducted in ICU has reported data on IRIS [10]. Among 278 HIV-infected patients admitted in ICU, three patients with tuberculosis developed IRIS during their ICU stay.

Our study is the first to report data on the use of HAART after the ICU stay. In our cohort, patients not receiving HAART in ICU initiated or restarted HAART after a median delay of three weeks after ICU release. This relatively short delay could have reduced the impact of HAART prescription in ICU. The optimal time to prescribe HAART in ICU patients (immediate treatment vs early treatment after ICU discharge) needs to be defined. The decision regarding when to administrate antiretroviral therapy in critically ill HIV-patients must balance the risk of HIV disease progression with the potential risk of drug toxicities. In our cohort, tolerance of HAART in ICU was good with only one patient (3%) suffering from a side effect motivating interruption of HAART. After ICU discharge, the incidence of adverse events was not different according to the timing of HAART prescription, during or after the ICU stay. Only one previous study conducted in ICU has reported data on HAART adverse effects. The incidence of side effects demanding either switch in or discontinuation of HAART was 18% [10]. Occurence of an adverse effect had no impact on mortality.

Another concern about HAART prescription in ICU is drug delivery. Most drugs are only available orally with few liquid medications, and absorption is often unpredictable in seriously ill patients. Multiple drug interactions with HAART can result in either toxic or subtherapeutic antiretroviral drug levels. In our cohort, we did not practice any drug monitoring. But we observed a higher proportion of antiretroviral drug resistance after ICU discharge in patients treated with HAART in ICU. Low antiretoviral drug concentrations in critically ill patients could lead to selection of resistant virus. Non compliance to HAART before ICU admission could have contributed to the resistance. All patients diagnosed with resistance after ICU have been exposed to HAART. But we did not specifically evaluate adherence to HAART.

Our study has several limitations in interpretation and the ability to generalize from its results. We conducted our study at a single institution. Clinical practice and demographics may differ across institutions, and countries. As in any observational retrospective study, the missing data could have reduced the strength of our observations. Because of the study’s retrospective nature, it is possible that patients receiving HAART differ in some systematic way beyond the direct effects of the medications. In our cohort, patients who received HAART in ICU were less likely to be ignorant of their HIV infection. Confounders may have affected our observations. But the major limitations of our study are the small size and heterogeneity of patients groups that prevent adequate statistical analysis. Nevertheless, the relatively short duration of inclusion time allowed standardized ICU care. All patients were admitted in ICU during an era including tight glycemic control, protective ventilation in case of acute respiratory distress syndrome, corticosteroids in case of *Pneumocystis* pneumonia or septic shock [21-23].

## Conclusion

In our cohort of critically ill HIV-infected patients, benefit of HAART administration during ICU stay was limited. The reticence of intensivists to use HAART and the early prescription of HAART after ICU discharge may have reduced the impact of HAART use in ICU. Our results support the need for prospective randomized clinical trials to address the optimal timing to prescribe HAART in critically ill patients. Future trials should provide antiretroviral drug monitoring and data on compliance to HAART after ICU discharge.

## Abbreviations

AIDS: Acquired immunodeficiency syndrome; CI: Confidence interval; HAART: Highly active antiretroviral therapy; HIV: Human immunodeficiency virus; ICU: Intensive care unit; IRIS: Immune reconstitution and inflammatory syndrome; NRTI: Nucleoside/Nucleotide reverse transcriptase inhibitor; PI: Protease inhibitor; OR: Odds ratio; SAPS: Simplified acute physiology score.

## Competing interests

The authors declare that they have no competing interests.

## Authors’ contributions

AM, LL have contributed to study design, analysis and interpretation of data. MV has performed statistical analysis. NV has contributed to interpretation of data and has revised critically the manuscript. NB, AC, HG drafted the manuscript. YY, OL have participated in data interpretation and manuscript drafting. All authors read and approved the final manuscript.
